# Peroral Endoscopic Myotomy (POEM) for Achalasia Cardia: A Report of Two Cases

**DOI:** 10.7759/cureus.77467

**Published:** 2025-01-15

**Authors:** Mukesh S Paudel, Dibas Khadka, Kumud Bhattarai, Bhupendra K Basnet, Ashish Agarwal

**Affiliations:** 1 Gastroenterology, National Academy of Medical Sciences, Kathmandu, NPL; 2 Gastroenterology, Bir Hospital, National Academy of Medical Sciences, Kathmandu, NPL; 3 Gastroenterology, All India Institute of Medical Sciences, Jodhpur, Jodhpur, IND

**Keywords:** achalasia cardia, first case from nepal, myotomy, peroral endoscopic myotomy (poem), third space endoscopy

## Abstract

Achalasia cardia is a motility disorder of the esophagus characterized by a lack of esophageal peristalsis and failure of lower esophageal sphincter opening during swallowing. Symptomatic patients present with a long history of difficulty swallowing, along with chest pain and vomiting.

Two male patients, 55 and 24 years old, with proven achalasia cardia by high-resolution esophageal manometry (HRM), underwent a successful peroral endoscopic myotomy (POEM) procedure at our center. This was the first time this procedure was done in Nepal. In this case report, we have demonstrated that a novel third-space procedure like POEM can be safely performed in a resource-limited country.

## Introduction

Achalasia originates from the Greek word a-khalasis, meaning lack of relaxation. Achalasia cardia is a motility disorder of the esophagus where there are abnormal contractions of the esophageal body with failure of relaxation of the lower esophageal sphincter (LES) during swallowing.

There are many treatments for achalasia cardia, including oral calcium channel blockers, endoscopic balloon dilation, botulinum toxin injection, surgical Heller’s Myotomy, and peroral endoscopic myotomy (POEM). In a resource-limited country like ours, balloon dilation has been the most common procedure for this disease [1]. However, few centers in the country are also equipped to perform laparoscopic Heller’s myotomy [2]. No facility in Nepal has provided POEM procedures for such patients.

In this case report, we report the outcomes of two patients with achalasia cardia who underwent POEM procedures for the first time in Nepal.

## Case presentation

Case 1

**Figure 1:**
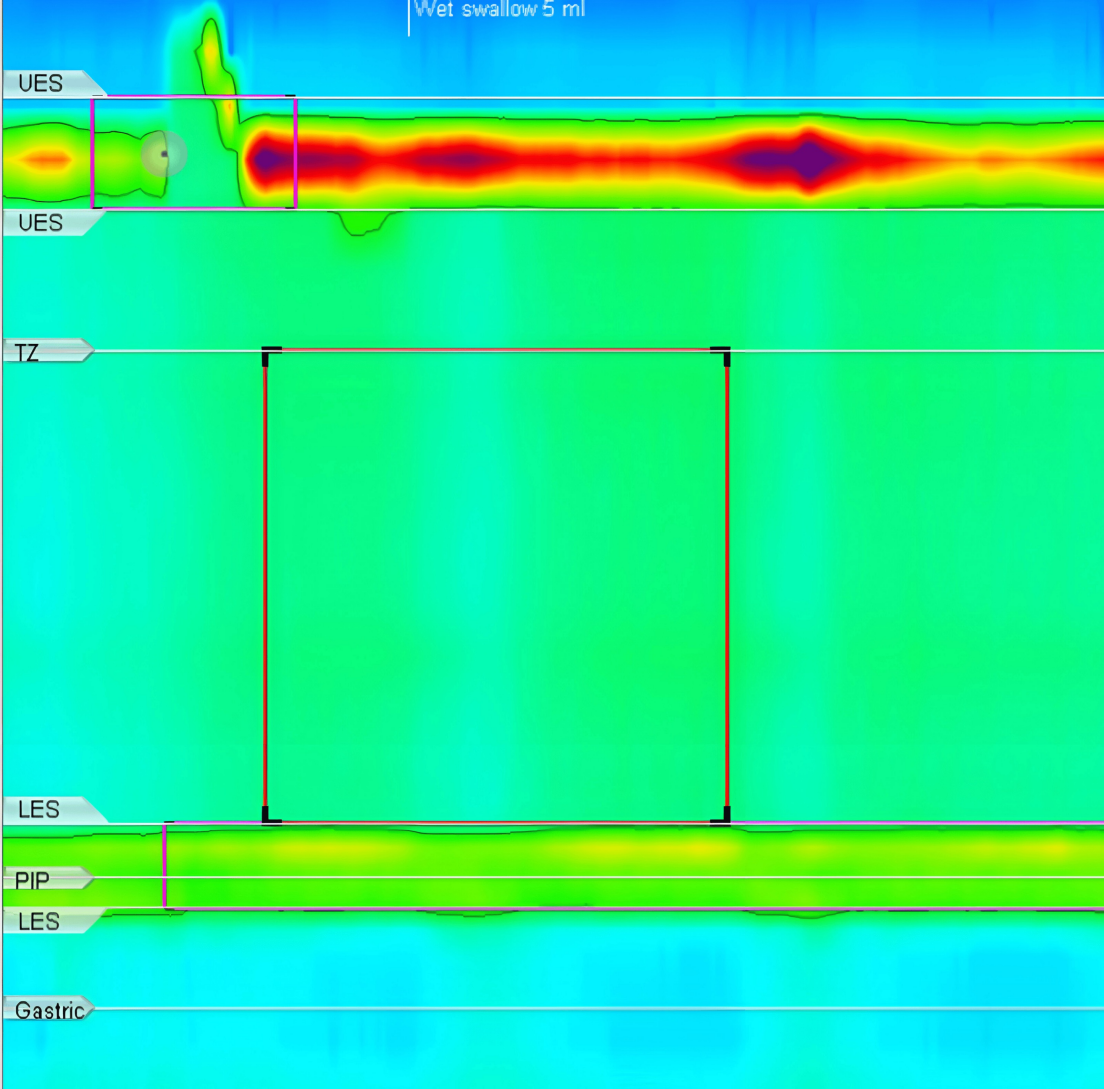
HRM image of case 1. HRM: high-resolution manometry, UES: upper esophageal sphincter, TZ: transition zone, LES: lower esophageal sphincter, PIP: pressure inversion point.

The first patient was a 55-year-old male. He complained of progressive dysphagia for the last 35 years, and his Eckardt's symptom score was five out of twelve. An endoscopic examination revealed a tight LES. Esophageal high-resolution manometry (HRM) examination showed a median integrated relaxation pressure (IRP) of 12.6 mmHg with all failed swallows (Figure 1).

**Figure 2:**
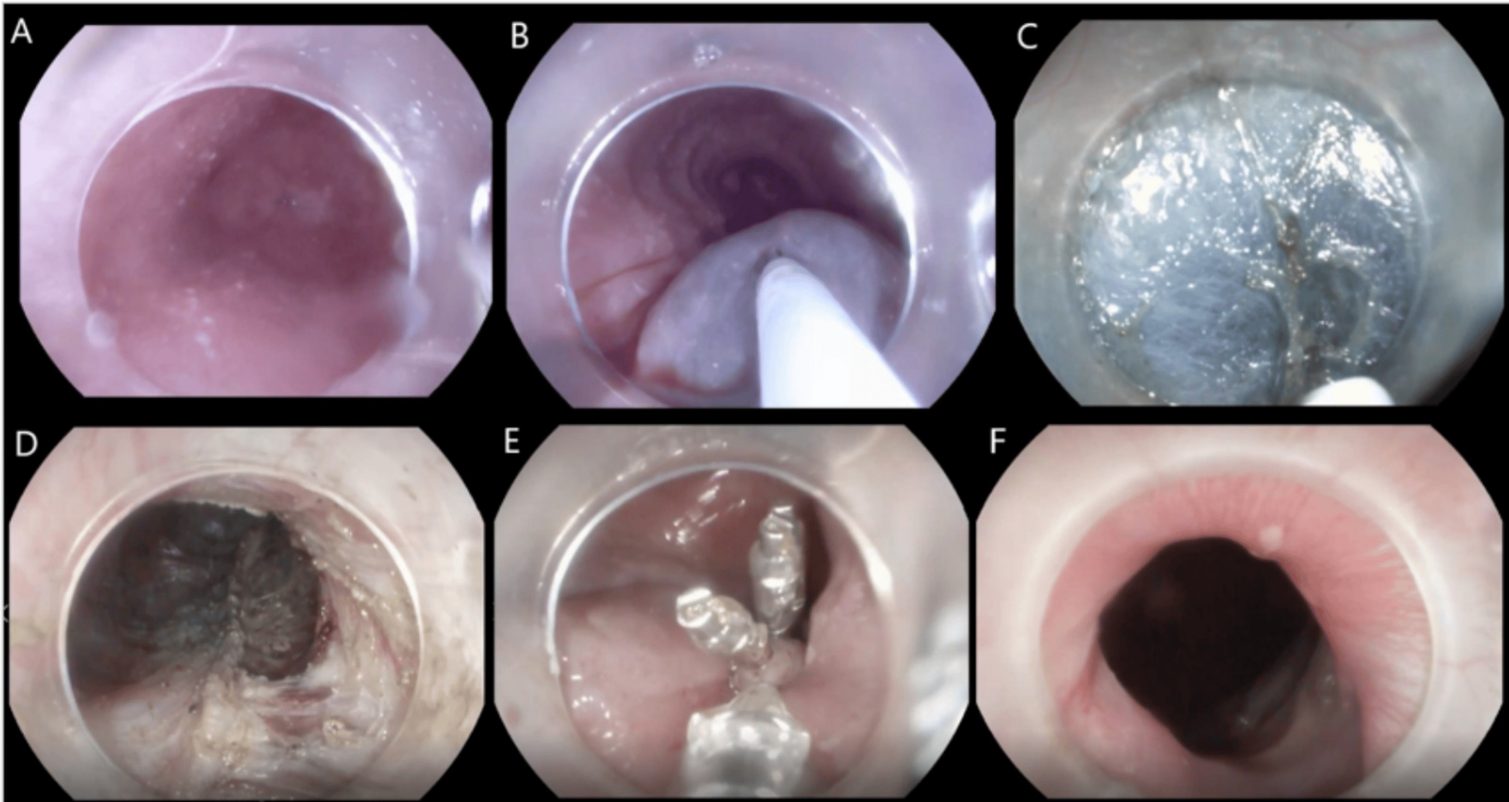
POEM procedure of case 1. (A) pre-POEM esophagogastric junction; (B) submucosal injection; (C) submucosal dissection; (D) myotomy; (E) mucosal defect closure; and (F) post-POEM esophagogastric junction. POEM: peroral endoscopic myotomy.

A diagnosis of achalasia cardia type 1 was made. The patient was advised to undergo a POEM procedure. The procedure was performed under anesthesia with a Pentax EG29i10 therapeutic gastroscope (Pentax Medical, Tokyo, Japan). A submucosal bleb was created with a solution of normal saline and indigo carmine approximately 10 cm above the gastroesophageal junction. A longitudinal incision of about 1.5 cm was made over the bleb, and a submucosal space was entered with the help of a gastroscope fitted with a transparent distal attachment cap. Submucosal fibers were dissected with a triangle-tip knife beyond the gastroesophageal junction. Circular muscle myotomy was done up to the lower esophageal sphincter and any vessels encountered during the procedure were coagulated with the help of a coagrasper. The mucosal incision was closed with the help of hemoclips (Figure 2).

The POEM procedure was done without any intra-procedural and post-procedural complications. A gastrografin swallow was done the next day, which did not show any leakage of contrast, and the patient was discharged with advice to take a liquid diet for one week. Because the patient lives in the far-western part of Nepal, an in-person follow-up was not possible. He was contacted via telephone after two months and reported a significant improvement in his symptoms with a gain in weight and an Eckardt score of one out of twelve.

Case 2

The second patient was a 24-year-old male with a seven-month history of dysphagia more to liquids than solids and had a weight loss of five kilograms. His Eckardt symptom score was eight out of twelve. HRM showed a median IRP of 24.4 mmHg with panesophageal pressurization.

**Figure 3:**
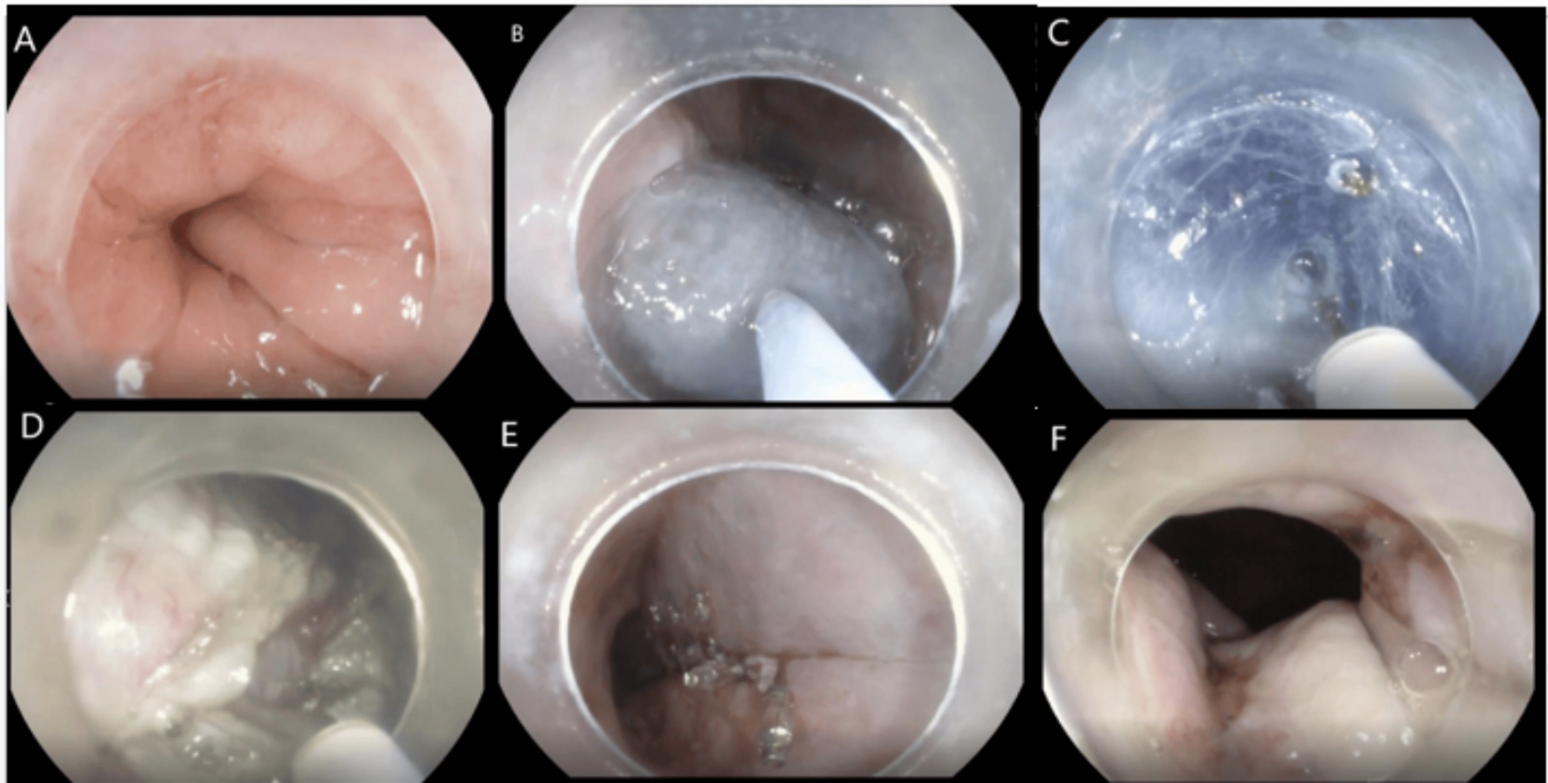
POEM procedure for case 2. (A) pre-POEM esophagogastric junction; (B) submucosal injection; (C) submucosal dissection; (D) myotomy; (E) mucosal defect closure; (F) post-POEM esophagogastric junction. POEM: peroral endoscopic myotomy.

A diagnosis of achalasia cardia type 2 was made. POEM was done following the steps outlined for case 1, and the patient was discharged the next day after his gastrografin swallow, which demonstrated no leakage (Figure 3).

His follow-up HRM done at three months after the procedure showed improvement with a median IRP of 4.3 mmHg. His post-procedure Eckardt score was zero out of twelve.

## Discussion

Achalasia cardia is a rare disease with a reported incidence of one per 1,00,000 population. Peroral endoscopic myotomy is a novel procedure for the management of achalasia cardia, which was first performed in Japan in 2008 [3]. A randomized controlled trial (RCT) has shown that laparoscopic hellers myotomy, compared to balloon dilation, is not associated with superior rates of therapeutic success in these patients [4]. POEM has also been shown to be non-inferior to the laparoscopic Heller’s myotomy with Dor’s fundoplication in patients with achalasia cardia [5].

The main symptoms of achalasia cardia are gradually progressive dysphagia, more to liquids than solids, weight loss, vomiting, and chest pain. These symptoms were present in both of our cases.

Diagnosis of achalasia relies on the symptoms, barium swallow examination, and endoscopy findings. High-resolution manometry measures the function and pressure of esophageal muscles. Chicago version 4 classification of esophageal motility disorders requires a high median IRP and either 100% failed peristalsis with ≥20% swallows showing pan esophageal pressurization or spastic/premature contractions [6]. It has been shown that patients with longer duration of achalasia symptoms and those who are old may have achalasia with normal IRP [7]. A normal IRP was seen in case 1.

POEM can be performed via either an anterior or posterior tunnel orientation, with comparable efficacy, safety, and rate of post-procedure reflux between these two approaches [8]. Both of our patients had undergone posterior tunneling. Post POEM, many patients may develop mild esophagitis with reflux symptoms; however, most of these can be managed by proton pump inhibitors [9]. Both of our patients received daily proton pump inhibitors for six weeks post-treatment and didn’t have reflux symptoms at the end of three months.

## Conclusions

Third-space endoscopic procedures like POEM have been labeled as the future of treating gastrointestinal dysmotility disorders. In this case report, we have described the first two cases of patients with achalasia cardia managed by POEM procedure in Nepal. The major hindrances faced in starting a POEM procedure in a limited resource setting are the rarity of the disease, the unavailability of manometry machines for the diagnosis of achalasia cardia, the unavailability of the standard equipment for the procedure, and the lack of trained manpower. Both POEM procedures were performed by the authors under the supervision of a trained expert. Future POEM procedures are planned to be done similarly and also on animal models. In this case report, we have demonstrated that even in a resource-limited country like Nepal, POEM can be performed safely.
